# Role of TRP channels in dendritic integration and subthreshold membrane potential plateaus

**DOI:** 10.1186/1471-2202-12-S1-P110

**Published:** 2011-07-18

**Authors:** Marcus E Petersson, Erik A Fransén

**Affiliations:** 1 Dept. of Computational biology, School of Computer Science and Communication; Stockholm Brain Institute, Royal Institute of Technology, AlbaNova University Center, Stockholm, SE-106 91, Sweden

## 

Cortical as well as subcortical neurons display plateau properties [1]. The plateaus may be voltage and/or calcium gated and depend on Na, Ca or mixed cation (TRP) currents. Depending on the nature of the synaptic input, neurons may respond by entering one of several multiple stable states, in the form of either subthreshold or persistent firing. We have studied the activation of a TRP current, in which synaptic excitatory input activates metabotropic glutamate receptors in turn leading to a TRP-mediated slow EPSP, both experimentally [2] and computationally [3]. In the computational work we have studied the contribution of the TRP current on dendritic integration of input of low (1-10 Hz) frequency.

The model is based on a CA1 pyramidal cell model [4] developed to study dendritic processing. The spatial compartmentalization had been obtained from a digitized neuron and contains 183 compartments. It includes ion channels described by a Hodgkin-Huxley formalism and has the following channels: Na, Na_P_, Ca_R_, Ca_T_, Ca_L_, K_dr_, K_A_, K_M_, K_(Ca)BK_, K_(Ca)slow_, K_leak_, h. To this model we added models for a metabotropic glutamate receptor as well as a TRP channel, the latter being adapted from a CAN channel model.

**Figure 1:**
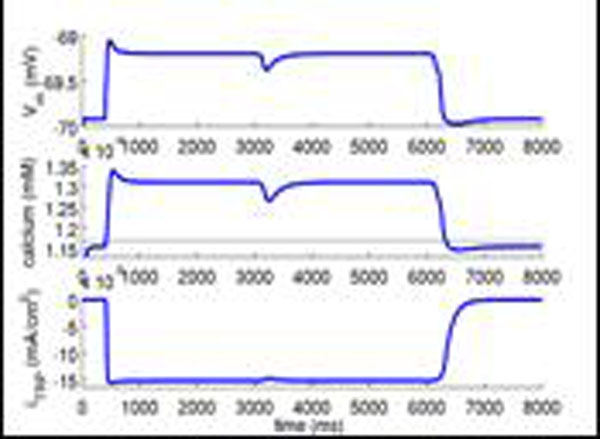
The membrane voltage (V_m_) is resting close to -70 mV. At the arrival of an excitatory synaptic stimulus, V_m_ enters a more depolarized, subthreshold plateau, which is stable to weak inhibitory input (t = 3000 ms). V_m_ returns to its resting state only if strong inhibitory input is applied (t = 6000 ms). This intrinsic upstate is due to the dynamical balance between the calcium concentration, V_m_ and calcium, TRP and outward currents.

Continuing our computational work, we have recently studied how the dendritic integration properties depend on the time constant of the calcium activating the TRP-current. In the model calcium comes from L-type Cav1.3 channels with a low threshold but there may also be contributions from T-type channels. We find that the effective integration time constant, the decay time constant of the neuronal membrane, increases when the amplitude of the calcium concentration increases. Moreover, recent experimental work [5] has shown that the decay time constant of calcium increases as a result of increased calcium concentration. This prompted us to study effects of adding a slower decay time constant in addition to the one present in the model used so far. Intriguingly, we find that a stable subthreshold plateau appears when a slower (200 ms) calcium decay time constant is present but not when only the original faster (30 ms) is present. This plateau of the resting potential is stable for weaker synaptic excitatory or inhibitory input, but stronger input can switch the membrane between the two levels, see figure 1. We find that the subthreshold plateau is essentially local to individual dendritic branches, and that the cell enters a persistent spiking [1] mode when several branches enters plateau mode. This bistability in membrane potential due to intrinsic conductances may be compared to the network driven changes of subthreshold membrane potential during cortical up and down states, suggested to switch the neuron between different modes of integration/operation.
